# Highlights from day three of the EuroSciCon 2015 Sports Science Summit

**DOI:** 10.4155/fso.15.12

**Published:** 2015-11-01

**Authors:** Amit Chawla, Alison McGregor

**Affiliations:** 1 Department of Surgery & Cancer, Faculty of Medicine, Imperial College London Charing Cross Hospital, London, UK

**Keywords:** conference, EuroSciCon, exercise, physiology, sports science

## Abstract

This EuroSciCon Sports Science Summit represented a significant gathering of leading professionals in the field of sports science. The conference was held on 13–15 January 2015 at the O2 arena, London, UK. The chairman on the third day was Mr Greg Robertson, a specialist trainee Orthopedic surgeon from Edinburgh. The conference attracted over 80 attendants from all over the world, with 32 presentations from invited speakers and peer-reviewed submissions. This meeting report provides a summary of the best abstracts from the conference.

Mr Greg Robertson presented the findings from a descriptive epidemiological study conducted by the Edinburgh Orthopedic unit. They investigated the epidemiological characteristics of sport-related fractures, their outcomes and the likelihood of return to sport after injury in the Lothian population during the period 2007 and 2008 [1–3].

A total of 990 fractures were recorded (incidence 1.8/1000/year), of which 75% involved the upper limb, and 23% lower limb, with 19% requiring surgical intervention. The median age of patients was 25 years, and 82% were male. The main sports implicated were football (35%), rugby (15%), cycling (11%) and alpine sports (11%). The group discovered that lower limb fractures took on average three-times longer than upper limb fractures, and operatively managed fractures took two-times longer than nonoperatively managed fractures to return to sport. They concluded that fractures should be properly assessed by experienced clinicians to determine whether they can be managed nonoperatively, as this can significantly affect clinical outcomes and return to sport.

Dr Wangdo Kim presented on behalf of the Faculty of Human Kinetics at the Technical University of Lisbon. They reported a novel surgical technique for ACL (anterior cruciate ligament) reconstruction to minimize the risk of graft impingement. Impingement occurs when the graft becomes trapped in the intercondylar notch, as a consequence of inaccurate positioning of the tibial tunnel, causing anterior knee pain, effusions and recurrent instability.

Dr Kim used James Gibson's ecological approach to locate the instantaneous axis of the knee during locomotion (an information algorithm as the basis for perception action control of the instantaneous axis of the knee). Dr Kim and colleagues hypothesized that muscle sensitivity is irrelevant for the perception of space and movement of the knee joint, but that the ACL plays an integral role. Based on this concept, they highlighted the theory of involution, which occurs when six lines acting upon the knee joint are situated such that the forces acting along them equilibrate. They ultimately proposed a surgical method to prevent impingement, based on the principle that the tibial tunnel can be placed in a manner that the force on each ACL graft is in involution with the original intact joint.

Dr Blaine Hoshizaki presented on behalf of the Neurotrauma Impact Science Laboratory, University of Ottawa, Canada. They reported on, ‘Mechanisms of head injury in sport and energy management characteristics of helmet technologies [4,5].' They alluded to four principal mechanisms of impact: falls, collisions, punches and projectiles. Impact event characteristics were described in terms of location, velocity, duration, mass and direction. It is clear that the mechanism of impact has an important influence on the risk of head injury. The location of the impact the angle of the force vector, the duration of the impact (compliance), impact mass and of course the impact velocity all influence the risk of head injury. Head injuries in sport are proving to be more complex than originally described. Dynamic response time curves resulting from impact to the head are influenced by the striking mass, compliance, impact velocity, impact locations and direction of force, and are specific to the types impacts experienced in specific sports. The relationship between the dynamic response of the head to impact and the risk of injury describe unique mechanisms of injury. These unique mechanisms of head injury described for each sport provide opportunities to develop more effective head protection in sport and physical activities.

Mr Xavier Gasparutto, on behalf of the Faculty of Human Movement Sciences, at the MOVE Research Institute, Amsterdam, reported on, ‘the role of pelvis and thorax rotation velocity in baseball pitching.’ This descriptive laboratory study aimed to examine the interaction of pelvis and thorax rotations in achieving high throwing velocities in pitching fastballs in a population of Dutch junior elite baseball pitchers. The literature has described associations between high segmental rotational velocities and high throwing velocities in baseball pitching, and suggests that the body works as a kinematic chain, in which segments are rotated in a sequential order. Mr Gasparutto and colleagues found that the relative timing of pelvis and thorax peak rotation velocity in pitching fastballs in baseball is a determinant of throwing velocity in skilled pitchers. They therefore concluded that separation of segmental peak rotations deserves to be focused on in scientific research as well as in developing training programs in baseball pitching. This study shows that, in addition to high segmental rotational velocities, the sequential timing of thorax and pelvis peak rotation velocity is associated with throwing velocity.

Dr Janne Avela presented on behalf of the Neuromuscular Research Centre, Department of Biology of Physical Activity, University of Jyvaskyla, Finland. They reported on, ‘Can material and structural modification of ice hockey arena dasher boards change impact characteristics of body checks.’ The study aimed to determine how ice hockey dasher board materials and structures affect impact characteristics and thereby concussion risk from body checks.

The group found that dasher board materials and structures had a major effect on impact characteristics. Flexible protective shielding material (Acryl) resulted in 17 and 16% lower peak forces, 110 and 136% greater stopping distances and 62 and 56% lower stiffness values compared with the reference dasher board (tempered glass). Single-framed dasher board was found to be 29 and 11% more flexible than its dual-framed counterpart, and heavier protective shielding resulted in 33 and 19% higher element stiffness in the straight and the corner parts of the dasher board, respectively. There were no significant differences in the impact characteristics of the stimulated body checks when laboratory data were compared with ice hockey arena data.

In light of the results, the group concluded that the safest dasher board would be single framed with light and flexible protective shielding material.

Julian Baker, an experienced physiotherapist and founder of Functional Fascia, described his method of anatomy teaching that focuses on the fascia and connective tissue structures, rather than standard approaches. Functional Fascia provides dissection-based anatomy teaching courses to health professionals and students, with a focus on learning about movement and function of body parts, and how they interact as a kinetic chain, rather than what Mr. Baker describes as the traditional rote method of memorizing Latin names of different structures. He also emphasized the importance of the superficial fascia, describing it as the translator of a therapist's touch to the deeper tissues beneath. His model states that the ability to treat deeper structures depends on how we manipulate the superficial fascia. Mr. Baker hopes that his teaching model will ultimately form the basis for anatomy teaching in undergraduate and postgraduate healthcare curriculams.

Dr Simon Marwood reported on behalf of the department of Health and Applied Social Sciences at Liverpool Hope University, on the topic, ‘the role of the tricarboxylic acid cycle intermediates (TCAi) in facilitating oxidative metabolism’.

At the onset of exercise there is an exercise intensity-dependent increase in the concentration of the TCAi, which reaches a peak after 10–15 min of exercise before declining. Since this phenomenon parallels the increased oxidative metabolism and thus TCA cycle flux, it has been suggested that the concentration of TCAi may be of functional importance for oxidative phosphorylation. Dr Marwood and his colleagues aimed to determine whether glutamine ingestion, which has been shown to enhance the exercise-induced increase in the TCAi pool size, resulted in augmentation of the rate of increase in oxidative metabolism at the onset of exercise. They focused on a subject group of chronic obstructive pulmonary disease patients, who have been shown to possess less muscle glutamate compared with nonsufferers.

The main finding from their study was that glutamine ingestion prior to exercise resulted in no beneficial effect on oxidative metabolism or performance during incremental and constant load exercise in chronic obstructive pulmonary disease. Peak work rate, peak oxygen uptake, oxygen uptake at lactate threshold and oxygen uptake kinetics remained unchanged following prior glutamine ingestion, compared with placebo. Considering these findings it is not surprising that blood lactate concentration and subjective indicators of fatigue were also unaffected by glutamine ingestion, and thus the significance of TCAi concentration in oxidative metabolism remains unclear.

Amit Chawla, a fifth year medical student, presented on behalf of the Department of Surgery and Cancer, Imperial College London. They reported on, ‘a novel approach to hamstring training: is it effective?' They described the theory of the peak torque angle of eccentric hamstring contraction acting as a risk factor for acute hamstring strain injuries during sprinting. This exploratory study sought to explore the impact of the Mujo hamstring training system, a novel piece of equipment with a biarticular mechanism, on the angle of peak torque during eccentric hamstring contractions in healthy individuals.

They found that no differences were elicited in the angle of peak torque post intervention with the Mujo trainer, compared with a control group with no intervention. Therefore, it is not clear whether this new biarticular training approach demonstrates any value to preventing hamstring injury. Its findings also question the robustness of the angle of peak torque as an outcome measure during an eccentric contraction.
